# Design of the Piano Score Recommendation Image Analysis System Based on the Big Data and Convolutional Neural Network

**DOI:** 10.1155/2021/4953288

**Published:** 2021-11-26

**Authors:** Yuanyuan Zhang

**Affiliations:** School of Art and Design, Qingdao University of Technology, Qingdao 266033, Shandong, China

## Abstract

In the era of big data, the problem of information overload is becoming more and more obvious. A piano music image analysis and recommendation system based on the CNN classifier and user preference is designed by using the convolutional neural network (CNN), which can realize accurate piano music recommendation for users in the big data environment. The piano music recommendation system based on the CNN is mainly composed of user modeling, music feature extraction, recommendation algorithm, and so on. In the recommendation algorithm module, the potential characteristics of music are predicted by the regression model, and the matching degree between users and music is calculated according to user preferences. Then, music that users may be interested in is generated and sorted in order to recommend new piano music to relevant users. The image analysis model contains four “convolution + pooling” layers. The classification accuracy and gradient change law of the CNN under RMSProp and Adam optimal controllers are compared. The image analysis results show that the Adam optimal controller can quickly find the direction, and the gradient decreases greatly. In addition, the accuracy of the recommendation system is 55.84%. Compared with the traditional CNN algorithm, this paper uses the convolutional neural network (CNN) to analyze and recommend piano music images according to users' preferences, which can realize more accurate piano music recommendation for users in the big data environment. Therefore, the piano music recommendation system based on the CNN has strong feature learning ability and good prediction and recommendation ability.

## 1. Introduction

Music is one of the popular entertainment media in the digital age. As the product of human creativity, music expresses thoughts and emotions in the form of sound, including melody, tone, and rhythm. There are many types of music, such as pop music, rock music, jazz, blues, and ballads. Scientific and technological progress has greatly enriched the genre and style of music. The types of music people create and listen to also vary from region to region and culture to culture. Different people have different tastes and preferences for music, and even the same person has different needs for music in different emotional states [1–3]. Recommendation system can be regarded as an information filtering system, which can extract important information fragments from a large number of dynamically generated information according to user preferences, interests, or observed behaviors to solve the problem of information overload [4, 5]. At present, image analysis recommendation system has a broader meaning, describing any system that inputs personalized recommendation. In the current era of big data, massive music data have brought new challenges to the effective application of the recommendation system. Therefore, how to obtain an effective recommendation system to detect individuals' interest in music and recommend music in line with their preferences is a hot issue in the current research.

The challenge of the music recommendation system is to establish a scheme that can constantly find attractive new music and understand users' music preferences. Generally speaking, music image analysis and recommendation system can be divided into three main parts: user, item, and user-item matching algorithm. Firstly, a user model can be established according to the user's age, gender, environment, lifestyle, and interest to distinguish the user's music taste. Secondly, the project composed of editing metadata, cultural metadata, and acoustic metadata can be used in various recommendation systems. Finally, personalized music is automatically recommended by the matching algorithm [6, 7]. The matching algorithm mainly includes two filtering methods, namely, collaborative filtering and content-based filtering. The obstacle of the collaborative filtering method is sparse evaluation matrix, which is usually uncertain because most users only see a small part of the music library. With the application of deep learning technology gradually extended to various fields, deep learning is more and more used by music recommendation systems. Deep neural network is mainly used to extract the audio signal or metadata of music tracks in music recommendation and learn the order of music tracks from the music playlist. As an effective deep learning method, convolutional neural network (CNN) has achieved good application results in the fields of image recognition and speech recognition [8, 9].

Among all digital music categories, piano music is favored by most listeners and performers. Therefore, this paper studies the recommendation of the piano score. Firstly, based on the theory of classical recommendation algorithm, an image analysis recommendation algorithm based on the CNN is proposed. Accordingly, the graphic representation method and feature extraction method of frequency domain features and note features of piano music are proposed, and the piano music image analysis and recommendation system based on the CNN classification is realized. The classifier based on the CNN is innovatively designed on the basis of user preference characteristics and further trained through note spectrum under different conditions, which is of great significance to improve the accuracy of the recommendation system.

This paper is divided into five parts. The first part expounds the research background that piano music is favored by most listeners and performers in the category of digital music. The second part describes the recommendation of the piano score and the research of related algorithms. The third part introduces the piano music recommendation system based on the CNN and expounds the personalized recommendation system under big data and the recommendation algorithm based on deep learning. It also studies the spectrum representation and feature extraction of piano music. The fourth part expounds the research results, compares the piano music classification results, and analyzes the CNN piano music recommendation algorithm. Finally, the full text is summarized.

## 2. Related Works

Music can relieve stress, and different music genres have different effects on people. Chang et al. [10] used a new clustering algorithm *K*-Means to perform the pregloss finish on music pieces to explore the automatic classification of music. Besides, the algorithm could provide music recommendation according to personal preferences through the personalized recommendation method based on collaborative filtering. They also stated that most of the existing music recommendation systems used content-based recommendation engines, which chose music only depending on users' historical preferences or music content, but neglecting users' emotions. Ayata et al. [11] proposed an emotion-based music recommendation framework which learned users' emotions from signals acquired by wearable physiological sensors. Abdul et al. [12] constructed an emotion-sensitive personalized music recommendation system to extract the correlation between user data and music. Moreover, they integrated users' emotions through physiological sensors and then used these physiological data to improve the performance of the recommendation engine. Kim et al. [13] adopted an integrated method of dynamic classification based on the long-term modulation spectrum and sequence classification based on the short-term spectrum to improve the music classification performance for tempo. They utilized the tempo-oriented music classification method to reflect user preference models to obtain higher user satisfaction. Zou [14] proposed an incremental Slope One algorithm considering the exponential growth of information resources in the big data environment, which had the advantage of adapting to the instantaneous change of data, greatly improving the performance of the recommendation system.

Deep learning has shown its superiority in computer vision, natural language processing, speech recognition, and other fields. In the field of music recommendation, most deep learning methods learn the user's temporal preference in light of the user's listening history. Wen [15] analyzed the multiscale feature extraction performance of the fast regions with CNN features' algorithm based on deep learning and designed an intelligent background music system based on deep learning and the Internet of Things. Zheng et al. [16] proposed a deep temporal neural music recommendation model based on music characteristics and user temporal preferences. They encoded music metadata into a thermal vector which was projected to the low-dimensional space by using the deep neural network to obtain the music feature. Da'u and Salim [17] believed that the problem of information overload had a serious impact on the recommendation of new media projects and utilized the CNN and recursive neural network (RNN) based on deep learning to effectively improve big data classification. Rosa et al. [18] proposed a knowledge-based recommendation system by combining the CNN with the bidirectional short-term memory network, which improved user satisfaction with the recommendation results. Schedl [19] stated that the piano score recommendation system should not only consider the user's general music preferences but also needs to consider personal playing preferences and playing skills to provide personalized recommendation.

In summary, the recommendation system has been widely used in many scenes in real life. Although many efforts have been made for the research work and concrete application of the personalized recommendation system, there are still some problems unsolved, such as data sparsity and cold start. Therefore, the user preference model is first constructed by a hidden semantic model matrix. Then, a piano music recommendation algorithm is proposed based on the CNN classifier. Finally, the classification and prediction of user preference music are realized according to the relationship between user behavior and music preference.

## 3. Piano Music Recommendation System Based on the CNN

### 3.1. Personalized Recommendation System under Big Data

People are gradually entering the era of information overload in the big data environment. Correspondingly, the recommendation system comes into being to help users get the needed information efficiently from the vast amount of information. The main task of the recommendation system is to contact users with information. On the one hand, it helps users find valuable information for themselves. On the other hand, it displays information in front of users interested in it, thus to achieve a win-win situation for information consumers and information producers. The recommendation system based on big data analyzes the user's historical records to understand the user's preferences, thus to actively recommend the information they may concern themselves with to meet the user's personalized needs. In a typical scenario, the Hadoop Distributed File System stores large amounts of customer data in multiple data nodes and a head node, also known as general nodes or edge nodes. Internal analysis software of memory will immediately take over the data once the data are loaded into the memory of the Hadoop cluster. The data nodes parallelly calculate the recommendation model and then return the best user project portfolio to the head node at the edge of the cluster for decision-making.

**Figure 1:**
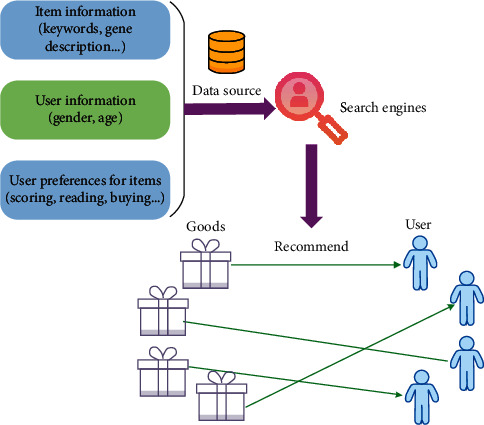
Working principle of the recommendation engine based on the user preference.

Figure 1 illustrates the working principle of the recommendation engine. User preference information can be divided into explicit user feedback and implicit user feedback. Explicit user feedback is the feedback information explicitly provided by users on or outside the site, such as user ratings and comments on items. Implicit user feedback is the data generated by the user when using the website, which implicitly reflects the user's preferences for items, such as information browsing or purchase records. Explicit user feedback can accurately reflect the user's real preferences for items, but it requires additional labor. On the contrary, implicit user behavior provides sketchy data concerning user preference through some analysis and processing, and there may be lots of noise in the analysis of some behaviors. Nevertheless, implicit user feedback can also achieve good results based on the premise of correct selection of behavior characteristics.

### 3.2. Recommendation Algorithm Based on Deep Learning

The traditional recommendation system mainly includes the following three methods: (1) recommendation system based on collaborative filtering: collaborative filtering algorithm provides related recommendations according to the similarity between users or between items from the perspective of similarity measurement, which commonly uses the memory-based method and model-based method. (2) Content-based recommendation system: unlike the collaborative filtering learning vectors from the interactive data of overall users and items, the content-based recommendation method learns the representation of users and items from the content of items. It believes that users may be interested in items like those they have previously interacted with. (3) Hybrid recommendation system: collaborative filtering method is prone to cold start or sparse data of the interaction matrix, while the hybrid recommendation system can use the information of users and items in the content-based recommendation system to alleviate this problem. The hybrid recommendation system can obtain better recommendation performance by integrating the content information of users and items, namely, auxiliary user information and auxiliary item information, into the collaborative filtering framework [20–22].

**Figure 2:**
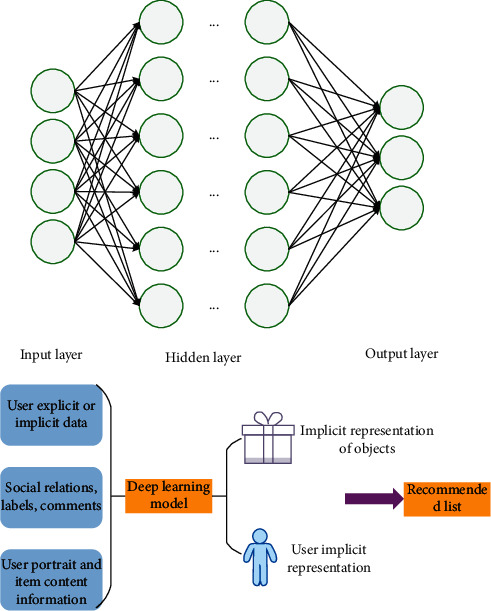
Structure of the recommendation system based on deep learning.

Deep learning is similar to the neural network simulating the human brain to perceive external stimuli and learn the essential characteristics of things from samples. The recommendation system based on deep learning can realize the representation of big data related to users and items by learning the deep network structure. Additionally, deep learning can capture the complex relationship between data through automatic feature learning and then complete the recommendation by combining with the traditional recommendation method to improve the cold start in the traditional recommendation system. Usually, the recommendation algorithm based on deep learning first takes the data related to users as the input, obtains the implicit representation of users by training the deep learning model, and then generates a targeted recommendation list according to this implicit representation.  Figure 2 represents the structure of the recommendation system based on deep learning.

Deep neural network contains many neurons and complex network connection methods, so the network has a strong advantage in computational performance, which can automatically extract high-dimensional features of data. Without extracting data sample features, the hidden layer in the deep learning network can automatically filter data and extract high-dimensional features.

### 3.3. Spectrum Representation and Feature Extraction of Piano Music

Piano music is usually stored in the form of digital audio information, which comes from the sampling of audio data and is also the most intuitive presentation of signals. The data size of audio depends on the sampling frequency, and a high sampling frequency indicates excellent audio fidelity. The time-domain characteristics of the signal can be discovered by expanding the digital audio information. Compared with the time-domain analysis of audio, the frequency-domain analysis can better reflect some substantive characteristics of audio [23, 24]. As a classical basic algorithm of frequency-domain analysis, the Fourier transform uses the sine and cosine function to fit the time-domain signal and utilizes the feature information carried by each sine and cosine function to represent frequency-domain features. The fast Fourier transform is an improved algorithm, which can significantly shorten the calculation time while maintaining the frequency-domain accuracy. Firstly, the continuous-time signal is transmitted through the ideal low-pass filter to filter out the high-frequency component higher than half of the sampling frequency to prevent the occurrence of spectrum aliasing during sampling. Then, the signal is sampled to obtain the discrete time signal.

The fundamental tone and overtone are the main elements of the piano music signal. Because the fundamental tone determines pitch, the key to the recognition of piano notes is the detection of the period of the fundamental tone. The fundamental tone detection based on the short-term autocorrelation function can be expressed as the following equation:

(1) $$\begin{array}{c} R_{i}\left(k\right)=\sum_{m-1}^{N-m}y_{i}\left(m\right)y_{i}^{\prime }\left(m+k\right). \end{array}$$

Equation (2) indicates the three-level center clipping before calculating the autocorrelation function:

(2) $$\begin{array}{c} y_{i}^{\prime }\left(x\right)=C\left[z_{i}\left(x\right)\right]=\left\{\begin{array}{cc} 1 & z_{i}\left(x\right)>C_{L}\\ 0 & \left|z_{i}\left(x\right)\right|\leq C_{L}\\ -1 & z_{i}\left(x\right)<-C_{L} \end{array}\right., \end{array}$$

where *C*_*L*_ represents the threshold level and *z*_*i*_(*x*) denotes the *i*th frame signal after the windowing and framing of the time series *w*(*t*) of the music signal.

Generally, the position corresponding to the first largest autocorrelation function is the period of the fundamental tone. The pitch frequency is the ratio of sampling frequency to the period of the fundamental tone, which has a congruent relationship with notes. The peak ratio is estimated through the data frame translation to select the correct peak point here. The data frame translation is achieved by adding the upper and lower bounds of the selected signal interval by 64, and the signal interval after translation can be written as the following equation:

(3) $$\begin{array}{c} seg\left(i\right)=w\left[S\left(i\right)+64,S\left(i\right)+len+64\right]. \end{array}$$

**Figure 3:**
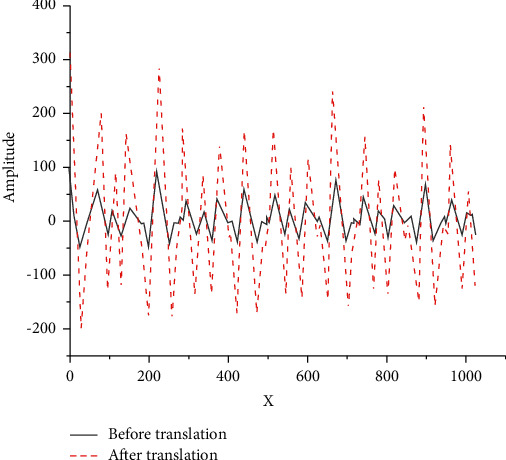
Amplitude comparison of the waveform frames of D5 notes before and after translation.

Figure 3 reveals the amplitude comparison of the waveform frames of D5 notes before and after translation. The peak value changes from 1.65 before translation to 1.35 after translation. Let *k* represent the constant of the peak ratio threshold, and count the number *n*_1_ of peak values greater than *k* and the number *n*_2_ of peak values less than *k* after 8 times of data frame translation. If *n*_1_<*n*_2_, the first peak point is taken as the period of the fundamental tone. Otherwise, set the maximum peak point as the period of the fundamental tone.

According to the basic theory of music, the relationship between note *i* and standard frequency *F*(*i*) can be expressed as the following equation:

(4) $$\begin{array}{c} F\left(i\right)=f_{a^{1}}*2^{n/12}. \end{array}$$

In equation (4), *n* means the number of semitones between note *i* and note *a*^1^, and *f*_*a*^1^_=440 presents the international pitch.

Equation (5) indicates the relationship between the period of the fundamental tone *T*(*i*) and the fundamental frequency *F*′(*i*):

(5) $$\begin{array}{c} F^{\prime }\left(i\right)=\frac{f_{s}}{T\left(i\right)}. \end{array}$$

In equation (5), *f*_*s*_ signifies the sampling frequency.

The cent deviation *O*(*i*) can be presented as equation (6), where $k=\sqrt[{1200}]{2}$.

(6) $$\begin{array}{c} O\left(i\right)=\;\log_{k}\left(\frac{F{}^{\prime }\left(i\right)}{F\left(i\right)}\right). \end{array}$$

Let *U*={*x|* − 50 < *x* < 50}, and when *O*(*i*) ∈ *U*, note *i* is correctly recognized.

### 3.4. Piano Music Classification and Recommendation System Based on the CNN

**Figure 4:**
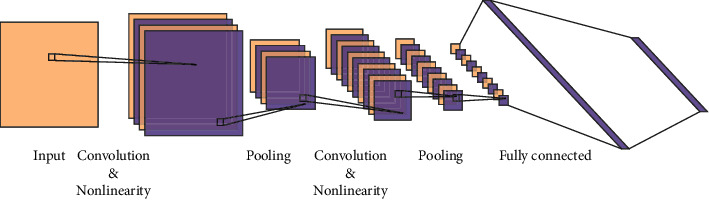
Architecture of the CNN.

As a representative algorithm of deep learning, convolutional neural network has the ability of representation learning; that is, it can extract high-order features from input information. Specifically, the convolution layer and pooling layer in the convolutional neural network can respond to the translation invariance of input features; that is, they can recognize similar features located in different positions in space. The ability to extract translation-invariant features is one of the reasons for the application of the convolutional neural network in computer vision problems. CNN is arranged in accordance with the image matrix and only connects with some neurons to significantly reduce weight parameters to avoid the defects of traditional neural networks such as oversized parameters and limited depth development of the network. CNN constructs a multilayer neural network by imitating the biological visual and perception mechanism. A typical CNN consists of three parts to convert the input into output, including the convolution layer, pooling layer, and fully connected layer [25–27]. These layers are stacked to form a CNN architecture, as shown in Figure 4. Each layer consists of multiple two-dimensional planes, and each two-dimensional plane contains several independent neurons. The convolution layer is the core part of the CNN architecture, characterized by a strong ability of feature learning. Usually, the first convolution layer can only extract relatively low-level features from the original data, while the deeper convolution layer can iteratively extract more complex features based on low-level features [28, 29]. Different from the fully connected network, CNN can share parameters for the same convolution kernel between the same layers, which effectively reduces the number of network parameters and can obtain more abundant structural features.

The proposed CNN classification model is implemented by the TensorFlow framework under the Windows 10, Python 4.0 system environment. The note spectrum of piano music is taken as the input image to train the CNN model under different conditions. The process of the piano music recommendation method based on the CNN can be divided into three steps. (1) Randomly separate music files into training samples and test samples, and segment the audio data to generate the spectrum feature map. (2) Compress the spectral characteristic map of training samples, and then put it into the CNN model for training to obtain the corresponding weights. (3) Classify test samples and recommend using the trained CNN model.

The pure piano performance audio files on https://www.hqgq.com are selected as the training sample of the CNN model, which are classified according to the criterion of classical, jazz, blues, and pop, with 100 files in each category. The frequency level of audio is compressed to 128 Hz, and the frequency data are mapped to 50 pixels per second. Four categories of audio data are segmented, finally obtaining about 6000 image samples. The classification features of four musical genres are as follows: classical has an obvious phenomenon of amplitude attenuation, which is partially of high frequency, strong rhythm, and relatively sonorous. Jazz has the most obvious amplitude changes and superposition effect, with a fast tempo. The amplitude attenuation of blues is not obvious, and the overall work is relatively smooth. The attenuation distribution of pop is uniform, and the frequency is generally low, indicating that the rhythm of pop is strong.

The training model is constructed based on LeNet-5, including four “convolution + pooling” layers and 1024 fully connected neurons. The bias is initialized to 0, and the learning rate is 0.001. The images are classified in the softmax layer after passing through two 2 × 2 convolution kernels, four “convolution + pooling” layers, and a fully connected hidden layer. Rectified linear unit (ReLU) is a very efficient activation function, which can be expressed as

(7) $$\begin{array}{c} f\left(x\right)=\max\left(0,wx+b\right). \end{array}$$

ReLU sets the output of some neurons as 0, improving the sparsity of the network, to reduce the mutual dependence between parameters.

When the CNN model is iteratively trained by the stochastic gradient descent method, it can extract a batch of samples to update the parameters. The loss function corresponds to the granularity of each sample. The iterative process is shown as

(8) $$\begin{array}{c} J\left(\theta_{0},\theta_{1}\right)=\frac{1}{2}{\left(h_{\theta }\left(x^{\left(i\right)}\right)-\left(y^{\left(i\right)}\right)\right)}^{2},\\ \theta_{j}=\theta_{j}-\alpha \frac{\partial }{\partial \theta_{j}}J\left(\theta_{0},\theta_{1}\right). \end{array}$$

Logistic function can only be applied to binary classification problems, but its polynomial regression can be applied to multiclass classification problems. Let the softmax function be *σ*(*z*) and the input *z* be a vector of dimension *C*; the output of the softmax function is also a vector *y* of dimension *C* (0 or 1).

(9) $$\begin{array}{c} y_{c}=\sigma {\left(z\right)}_{c}=\frac{e^{z}c}{\sum_{d=1}^{C}e^{z}d},\\ \overset{C}{\underset{c=1}{\sum }}y_{c}=1,\\ \overset{C}{\underset{c=1}{\sum }}y_{c}=1. \end{array}$$

Softmax is the output layer of the CNN model, and *C* neurons in the function represent *C* classifications. Since the total probability of each classification is 1, every category classified by the softmax function is mutually exclusive.

Users' preferences can be obtained combined with the relationship between piano music and users and based on the relationship between piano music and classification characteristics. Among the 1000 piano music audio files, 100 are from each user, and 1000 classification features can be obtained by the classification and prediction through the trained CNN. The classification model based on hidden Markov is used to calculate the user's music list to make the prediction results closer to the user's behavior habits.

The piano music recommendation system based on the CNN mainly includes user modeling, music feature extraction, and recommendation algorithm. Firstly, the historical music behavior data of users in the system are collected to construct the user preference feature model. Next, the audio content is preprocessed, and the spectral characteristics are extracted to continuously train the CNN model to obtain the regression model for predicting the potential characteristics of music. In the final recommendation algorithm module, the regression model is used to predict the potential characteristics of music, and the matching degree between users and music is calculated according to user preferences. The model generates the piano scores that the user may be interested in, sorts them, and finally recommends the new piano music to the relevant users.

## 4. Results and Discussion

### 4.1. Comparison of Classification Results of Piano Music

**Figure 5:**
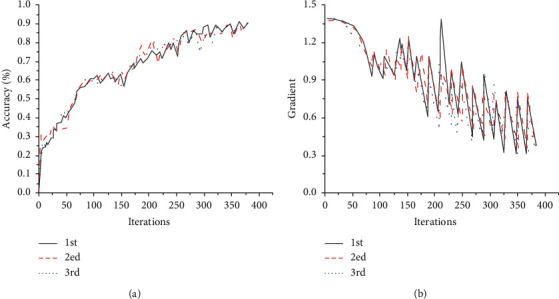
Experimental results by the ReLU activation function under the learning rate of 0.001. (a) Classification accuracy. (b) Gradient change.

The spectral characteristic maps are selected as training samples and test samples to verify the training effect of the CNN model under different conditions and count the classification results. Due to the strong randomness of neural network training, there will be bias in each classification result. Firstly, three repeated experiments are conducted under the identical learning rate, activation function, and optimal controller of the neural network, and the results are shown in Figure 5. The statistical results indicate that there are some differences in the accuracy and gradient changes of each experiment, but the overall trend is roughly the same. The difference between the final classification accuracy of different experiments is less than 0.05.

**Figure 6:**
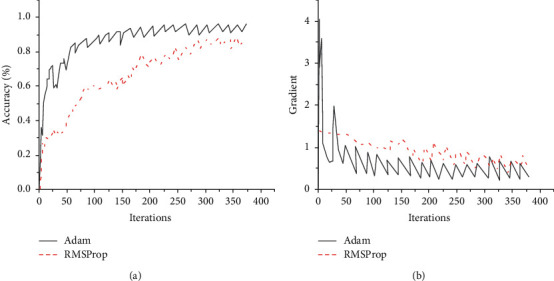
Comparison of experimental results under different optimal controllers. (a) Classification accuracy. (b) Gradient change.

Next, the classification accuracy and gradient variation rhythm of the CNN model are compared under two optimal controllers of RMSProp and Adam. According to the experimental results in Figure 6, the Adam optimal controller has better gradient descent ability and decline rate than RMSProp. In addition, combined with the trend of gradient variation, after about 50 times of training by huge fluctuation, the Adam optimal controller can quickly find the direction, and the gradient decreases greatly and quickly remains stable afterwards.

**Figure 7:**
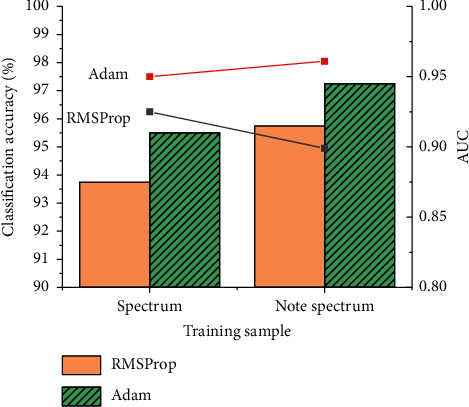
Comparison of training results by samples of the spectrum and note spectrum.

When the learning rate is 0.001, the CNN model is trained by using spectrum and note spectrum samples under the condition of the gradient descent method based on two optimal controllers. The test results are shown in Figure 7, which demonstrate that, under the identical condition, the training results of using the note spectrum as the training sample are significantly better than those of using the spectrum as the training sample. Under the RMSProp and Adam optimal controllers, the classification accuracy is increased by 2.0% and 1.25%, respectively. Therefore, the note feature can improve the classification effect of the CNN model on the spectrum to a certain extent.

### 4.2. Analysis of the CNN Piano Music Recommendation Algorithm

**Figure 8:**
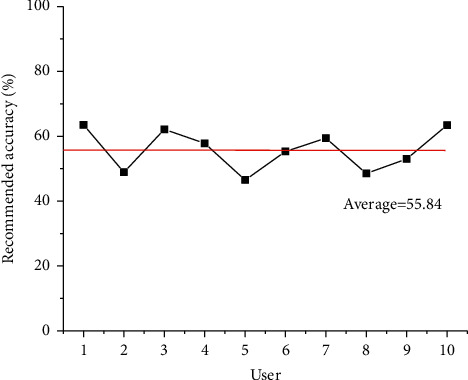
Recommendation results based on user characteristic evaluation.

According to the experimental results of the recommendation algorithm for comprehensive evaluation based on user characteristics, the average preference characteristic of users is calculated, and the preference list of each user is the reference sample generated by the hidden Markov model.  Figure 8 illustrates the recommendation results based on user characteristic evaluation, proving that the recommendation accuracy of single-classified users is higher than that of multiclassified users in the average preference feature. The main reason is that there is a little cross-genre piano music, leading to a small probability of random acquisition. The classification features used in the CNN model only contain music spectrum features, which can be integrated with text features of music, such as titles and descriptions, to form more accurate music features.

## 5. Conclusion

In the era of big data, the explosive growth of online information significantly increases the complexity and variability of the data content. Original information retrieval methods fail to meet the new needs of users due to serious information overload. Recommendation system is an effective information filtering tool which is widely used because of the increase in internet access, the trend of personalization, and the change of computer users' habits. Introducing deep learning into the recommendation system can improve the recommendation quality.

The CNN model can extract potential factors from audio, text, and image data to raise users' interest in recommendation when potential factors cannot be obtained from the user feedback. On this basis, a piano music recommendation system based on the CNN is constructed after clarifying the method of spectrum representation and feature extraction of piano music. The piano music recommendation system needs labelled sample datasets to facilitate learning in the early stage of the model. In other words, it is necessary to input a batch of labelled recommendation data for learning and calculation so that the CNN model can accurately recommend piano music. Combined with music classification and user preference characteristics, a music recommendation method is proposed based on the comprehensive evaluation of user characteristics. Moreover, the pure piano performance audio on https://www.hqgq.com is selected as the training sample to test the performance of the CNN recommendation system. The training results with the note spectrum as the training sample are significantly better than those with the spectrum as the training sample. Under the RMSProp and Adam optimal controllers, the classification accuracy is increased by 2.0% and 1.25%, respectively. Besides, the accuracy rate of the recommendation method reaches 55.84%, which is better than that of similar international methods. Compared with the traditional CNN algorithm, this paper uses the convolutional neural network (CNN) to design a piano music image analysis and recommendation system based on the CNN classifier and user preference, which can realize accurate piano music recommendation for users in the big data environment and has higher recommendation accuracy. The modules and process of the music recommendation system based on the CNN are roughly completed, but the system fails to fully combine the user's interest intensity of music. The users' records of share, like, and favour are well worth considering to build a more accurate music preference model.

## Data Availability

The data used to support the findings of this study are available upon request to the author.
